# Correction to: Regulating community well-being through traditional mourning rituals: Insights from the Luhya People of Kenya

**DOI:** 10.1093/emph/eoaf006

**Published:** 2025-03-11

This is a correction to: Stephen Asatsa *et al.*, Regulating community well-being through traditional mourning rituals: Insights from the Luhya People of Kenya, *Evolution, Medicine, and Public Health*, Volume 13, Issue 1, 2025, Pages 14–24, https://doi.org/10.1093/emph/eoaf001

The following change has been made to the original publication. The **Funding** section has been updated to clarify the status of grant support from the John Templeton Foundation.

